# Association between energy drink consumption, and substance use among adolescents: a cross sectional study

**DOI:** 10.3389/fnut.2025.1709218

**Published:** 2025-12-05

**Authors:** Maisa Nabulsi, Muna Ahmead

**Affiliations:** 1 Faculty of Pharmacy, Al-Quds University, Jerusalem, Palestine; 2 Faculty of Public Health, Al-Quds University, Jerusalem, Palestine

**Keywords:** energy drinks, alcohol, smoking, depression, adolescents, Palestine

## Abstract

**Background:**

Energy drinks (ED), which contain high doses of caffeine, are becoming increasingly popular, particularly among young people who may be unaware of the potential risks involved. The purpose of this study was to determine the prevalence of energy drink consumption among 12th-grade students, as well as the relationship between it and substance use, smoking, alcohol, depression, and anxiety.

**Methods:**

The study utilized a cross-sectional research approach. Data was collected using a self-reported questionnaire that included sociodemographic data, substance use-related questions, smoking and drinking factors, and the Hospital Anxiety and Depression Scale (HADS).

**Results:**

A total of 1,083 12th-grade students were recruited, revealing a high prevalence of energy drink consumption among them (52.5%). Also, the findings showed that participants whose father’s education was more than 12 years (AOR: 1.652, *p* < 0.001), participants who smoked cigarettes (AOR: 2.240, *p* < 0.004), participants who drank alcohol (AOR: 2.374, *p* < 0.005), and students who used waterpipe tobacco smoking (AOR: 5.401, *p* < 0.001) were more likely to drink energy drinks. No association was found between energy drinks and substance use.

**Conclusion:**

Our study found that energy drink consumption is high among 12th-grade students, particularly those who smoke cigarettes, use waterpipe tobacco, or drink alcohol. Our findings highlight the pressing need to raise youth awareness and educate them about the negative health consequences of energy drink consumption.

## 1 Introduction

The phrase “energy drink” refers to a group of drinks without alcohol that include caffeine, guarana, taurine, herbs (such as *Ginkgo biloba*), and vitamins (1). In the past two decades, energy drink use has risen rapidly, particularly among young people and teens. According to one Canadian study, 34.1% of students used energy drinks (2). Also, Kaldenbach et al. (3) reported that 2.9% of youth used energy drinks on a regular basis, 52.3% had used energy drinks previously, and 44.7% had never used them (3). In addition, Zucconi et al. (4) found that in a survey of 16 European countries, 68% of teenagers had drunk an energy drink in the previous year (4). There is a widespread belief that caffeinated beverages, such as energy drinks, can enhance both mental and physical performance (5) because energy drinks include a high concentration of caffeine, which has the desired benefits of enhancing memory, boosting alertness, and elevating mood (6).

Nevertheless, there are serious health risks associated with such high consumption rates (5, 7). For example, they contain a lot of sugar or synthetic sweeteners, along with minerals, amino acids, and other chemicals and stimulants (8–10). These stimulants increase the production of neurotransmitters that are associated with pleasure and rewards, such as dopamine and norepinephrine. Such substances can lead to a sense of pleasure and mental wellness, which can contribute to addiction (8). According to De Giorgi et al. (11), there are additional health problems that might arise, such as problems with the cardiovascular system, gastrointestinal system, anxiety, sleeplessness, psychomotor agitation, and agitation (11), and might pose a greater threat of developing obesity and type 2 diabetes (12). On the other hand, studies conducted by other researchers have not shown any harmful impacts or implications on health (5, 13, 14).

Moreover, previous studies have related teenage energy drink intake to substance use, such as alcohol, cannabis, tobacco cigarettes, and non-medical prescription drug use (2, 9, 15, 16). In a research of Canadian students, Azagba et al. (17) revealed that energy drink users were more likely than non-users to participate in risky behaviors such as using tobacco, alcohol, marijuana, and other drugs and substances (17). Similarly, Terry-McElrath et al. (18) found a substantial association between the frequency of energy drink usage among American youths and the frequency with which drugs such as marijuana, alcohol, cigarettes, and amphetamines were taken in the previous 30 days (18). According to Leal and Jackson’s (19) research, 24% of high school seniors consume energy drinks, 71% have experimented with soft drugs, and 19% have taken hard drugs (19).

Additionally, energy drink consumption has been linked to various mental health problems and diseases. Many studies have found a rise in depression and anxiety symptoms, as well as energy drink consumption, among teenagers (3, 20). According to Kaldenbach et al.’s (20) study, teenagers who drank energy drinks were more likely to experience severe depression symptoms, and 20% of the overall group reported having severe depressive symptoms. Also, 16.3% of individuals who never used energy drinks had severe depressive symptoms, compared to 19.1% of those who used energy drinks sometimes and 34.6% of those who used energy drinks on a daily basis. Also, they found that 2.9% of teenagers drank energy drinks on a daily basis, whereas 52.3% had ingested any energy drinks. 18.3% of the overall group showed severe depressive symptoms (3). Furthermore, Richards and Smith’s (21) systematic review indicated a positive association between anxiety, depression, and energy drinks (21).

Palestine, like other countries, has increased its marketing of energy drinks, and there are no legislative limitations on who may purchase energy drinks. These energy drinks are prevalent in Palestinian society. For example, one study of Palestinian students in sixth and ninth grades found that about 12% used energy drinks in sixth grade and 20% in ninth grade (22). Another study, conducted by Qtait and Alarab (23), showed that 67.7% of Palestinian university students in a Polytechnic University drank energy drinks (23). However, there is a shortage of studies among students in 12th grade. The Palestinian education system is organized in a 12-years structure that comprises grades 1–12. After 12 years of formal schooling, twelfth-grade students must pass the Tawjihi (General Secondary Education Certificate Test), a national exam administered by Palestine’s Ministry of Education and Higher Education. This test is a key benchmark in the Palestinian educational system. It provides a comprehensive assessment of students’ qualifications for future university education and prospective career pathways. As a result, teachers and families and teachers put immense pressure on students to study hard to get excellent scores on exams (24). These students are more likely to have anxiety symptoms, excessive worry, impatience, attention problems, depression, and suicidal ideation (25). Energy drink consumption among high school students, particularly in the 12th grade, is of particular concern because this young population is engaged in academic pursuits and is an ideal target for promoters of energy drinks, which promise to boost energy, promote wakefulness, increase alertness, and improve mental and physical performance. Thus far, little research has been conducted into patterns of energy drink consumption within this age range and their relationship with substance abuse. The purpose of this study was to assess the prevalence of energy drink intake among 12th-grade students, as well as its relationship with socioeconomic variables, substance use, alcohol intake, smoking, depression, and anxiety. Additionally, it sought to assess the factors that predict the consumption of energy drinks among 12th-grade students.

## 2 Materials and methods

### 2.1 Participants, design and setting

The study was a descriptive cross-sectional survey performed from February 21 to May 1, 2025. It targeted all Palestinian twelfth-grade students from the West Bank and Jerusalem, including boys and girls aged 18 years old. The exclusion criteria included students who had any conditions that would prevent them from completing the study’s questionnaires such as severe depression or anxiety. Using a 0.05 level of significance, a 95% confidence level, and a 3% accuracy, the study utilized SurveyMonkey to calculate a sample size of 1,046 students as follows:


$$\mathrm{Sample}\,\mathrm{size}=\frac{\frac{Z^{2}\times p\,\left(1-p\right)}{e^{2}}}{1+\left(\frac{Z^{2}\times p\,\left(1-p\right)}{e^{2}\,N}\right)}$$

1. *N* = population
2. *e* = margin of error (percentage in decimal form)
3. *z* = z-score* (how many standard deviations data is from the mean)
*95% confidence level is a 1.96 z-score

However, a total of 1,083 students filled out the questionnaire. The participants were recruited using convenience sampling, a non-probability approach that selects people from the target population based on their availability. An anonymous online self-administered survey was used to collect data. Due to the Israeli military’s blockade on Palestinian cities and travel restrictions in the West Bank, the researcher sent an electronic version of the questionnaire to the students using Google Forms, along with an introductory invitation. The researcher distributed the questionnaire online using Facebook student groups, social media sites, and WhatsApp messages.

### 2.2 Measures

Participants in this study completed a self-reported questionnaire with 3 sections. Section one included a socio-demographic sheet that included the participant’s gender, place of residence, father’s and mother’s education, and work.

Second section had energy drinks and substance use questions. Energy drink consumption was assessed by the following 2 questions: During the past month, did you consume energy drinks? Response categories were 1 = “yes” and 2 = “no.” 2). The other question was: How many times did you drink a can, bottle, or glass of caffeinated energy drinks such as Monster, Red Bull, XL, Full Throttle, or Rock Star?” Response categories ranged from 1 = Every day or almost every day, 2 = More than 3–4 times per week, 3 = More than 3–4 times per month, 4 = Once per month, 5 = Once per week. Cigarettes smoking was assessed with the following question: “Have you tried cigarette smoking in the last year? Response categories were 1 = “yes” and 2 = “no.” 2). Further, waterpipe tobacco smoking was assessed with the following question: “Have you tried waterpipe tobacco smoking in the last year? Response categories were 1 = “yes” and 2 = “no.” 2). Also, alcohol consumption was assessed with the following question: “Have you ever had a drink of alcohol in the last year?” Response categories for 1 and 2 were 1 = “yes” and 2 = “no.” Additionally, the use of other drugs or substances was assessed with the question, “Have you used any of the following drugs or substances in the last year: marijuana, crack, cocaine, hashish, heroin, Ecstasy, sedatives, sniffed glue, breathed the content of spray cans, or inhaled any paints or sprays to get high, without a doctor’s prescription?” Response categories were coded with 1 = “yes” and 2 = “no.”

The third section had the Hospital Anxiety and Depression Scale (HADS), which is a 14-item scale created to assess the presence of anxiety and depression. The HADS creates two scales to distinguish the two states: HADS–A for anxiety (seven questions) and HADS–D for depression (seven questions). On a 4-point severity scale, items are rated, and each question is scored between 0 (no impairment) and 3 (severe impairment), with 3 denoting the highest anxiety or depression level. A case is considered conclusive if the score on either scale is greater than or equal to 11. A score of 0–7 is considered normal, 8–10 indicates mild depression, 11–14 indicates moderate depression, and a score of 15–21 is equal to severe depression. The internal consistency coefficient (Cronbach’s α) was 0.825.

A committee of five mental health specialists reviewed the scale’s content to ensure cultural appropriateness, and no changes were made. The scale was first translated into Arabic by the study team and then back into English by a professional translator. During the pilot phase, the instrument was administered to 25 students to assess language clarity; both the original English questionnaire and the back-translated version were reviewed to ensure translation correctness.

### 2.3 Statistical analysis

The data were analyzed with SPSS version 25 (IBM Corp., Chicago, IL, USA). The descriptive analysis for all study variables is reported in the form of frequencies and percentages, and a chi-square test was performed. Furthermore, a multivariate regression analysis was carried out, and the results were given as adjusted odds ratio (AOR) with a 95% confidence range. Phi and Cramér’s V tests were used to assess the strength of the relationship between energy drink consumption and study variables. The adjusted model included all potential study confounders as well as factors associated with energy drinks. A *p*-value of less than 0.05 was considered a significant association.

### 2.4 Ethics

This study adhered to the Declaration of Helsinki in all its methods. The study was approved by Al-Quds University Research Ethics Committee (Ref No: 431/REC/2024). This online survey was anonymous. At the beginning of the study, written information about the aim of the study and how the data would be used was provided. Also, students were asked to obtain permission from their caregivers or parents before filling out the questionnaire. Upon filling out the questionnaire, students provided informed consent for participation in this study. Upon filling out the questionnaire, students provided informed consent for participation in this study.

## 3 Results

### 3.1 Socio-demographic characteristics of the participants

Table 1 shows that the majority of participants (67.8%) were female, 48.6% lived in cities, 62.6% of participants’ fathers had less than or equal to 12 years of education, and 53.8% of participants’ mothers had less than or equal to 12 years of education. Furthermore, 66% of respondents said their mothers were jobless.

**TABLE 1:** Socio-demographic characteristics of participants.

Characteristics		*N*	%
Gender	Male	349	32.2%
	Female	734	67.8%
Place of residency	City	526	48.6%
	Village	483	44.6%
	Camp refugee	74	6.8%
Father’s work	Unemployed	156	14.4%
	Employed	248	22.9%
	Private work	400	36.9%
	Workers (daily paid)	279	25.8%
Mother’s work	Unemployed	715	66.0%
	Employed	263	24.3%
	Private work	105	9.7%
Father’s education level	>12 years	405	37.4%
	≤12 years	678	62.6%
Mother’s education level	>12 years	500	46.2%
	≤12 years	583	53.8%

Table 2 shows that 9.0% of the participants smoked cigarettes, 6.5% drank alcohol, 52.5% consumed energy drinks, and 20.3% used pipe water. Also, 1.3% of the participants used substances such as marijuana, crack, cocaine, hashish, and heroin.

**TABLE 2:** Prevalence of smoking, alcohol and substance use in study participants.

Characteristics		*F*	%
Did you smoke cigarettes in last year?	Yes	98	9.0%
	No	985	91.0%
Did you drink alcohol in last year?	Yes	70	6.5%
	No	1013	93.5%
Do you drink energy drinks in the past month?	Yes	569	52.5%
	No	514	47.5%
During the past month how many times did you drink a can, bottle, or glass of caffeinated energy drinks such as Monster, Red Bull, XL, Full Throttle, or Rock Star?	Every day or almost every day	108	10.0
	More than 3–4 times per week	153	14.1
	More than 3–4 times per month	308	28.4
Did you use waterpipe tobacco smoking in last year?	Yes	220	20.3%
	No	863	79.7%
Did you take any drugs or substance in last year (e.g., marijuana, crack, cocaine, hashish, and heroin… etc.?)	Yes	14	1.3%
	No	1069	98.7%

### 3.2 The association between energy drink, sociodemographic variables, smoking, alcohol and substance use

Table 3 shows the results of the chi-square test, which examined the associations between energy drinks, respondent characteristics, and all study factors. Significant weak relationships were found between energy drink consumption and father’s work (*p* < 0.018*) and father’s education level (*p* < 0.016*). Furthermore, a strong significant relationship was found between energy drinks consumption and smoking cigarettes in the last year (*p* < 0.001), and a moderate significant relationship was found between energy drink consumption and drinking alcohol (*p* < 0.001). Finally, a very strong significant relationship was found between energy drink consumption and waterpipe tobacco smoking (*p* < 0.001).

**TABLE 3:** The association between energy drink, sociodemographic variables, smoking, alcohol, substance use, depression and anxiety.

		Energy drink					
		Yes (*n* = 569)		No (*n* = 514)		*P*-value	Phi and Cramér’s V
Characteristics		*N*	%	*N*	%		
Gender	Male	182	32.0%	167	32.5%	0.859	0.005
	Female	387	68.0%	347	67.5%		
Type of your school	Governmental	483	84.9%	426	82.9%	0.806	0.027
	Private	86	15.1%	88	17.1%		
Academic stream	Science	259	45.5%	223	43.4%	0.465	0.083
	Art	270	47.5%	245	47.7%		
	Others	40	7.0%	46	8.9%		
Place of residency	City	272	47.8%	254	49.4%	0.813	0.020
	Village	259	45.5%	224	43.6%		
	Camp refugee	38	6.7%	36	7.0%		
Father’s work	No work	66	11.6%	90	17.5%	0.018*	0.096
	Employees	141	24.8%	107	20.8%		
	Private work	221	38.8%	179	34.8%		
	Workers	141	24.8%	138	26.8%		
Mother’s work	No work	391	68.7%	324	63.0%	0.143	0.060
	Employees	127	22.3%	136	26.5%		
	Private work	51	9.0%	54	10.5%		
Father’s education level	>12 years	232	40.8%	173	33.7%	0.016*	0.073
	≤12 years	337	59.2%	341	66.3%		
Mother’s education level	>12 years	264	46.4%	236	45.9%	0.874	0.005
	≤12 years	305	53.6%	278	54.1%		
Did you smoke cigarettes in last year?	Yes	78	13.7%	20	3.9%	<0.001*	0.171
	No	491	86.3%	494	96.1%		
Did you drink alcohol in last year?	Yes	53	9.3%	17	3.3%	<0.001*	0.122
	No	516	90.7%	497	96.7%		
Did you use waterpipe tobacco smoking in last year?	Yes	183	32.2%	37	7.2%	<0.001*	0.310
	No	386	67.8%	477	92.8%		
Did you take any drug substance in last year (e.g., marijuana, crack, cocaine, hashish, and heroin… etc.?)	Yes	9	1.6%	5	1.0%	0.376	0.027
	No	560	98.4%	509	99.0%		
HADs Depression Scale	≤10	409	71.9%	378	73.5%	0.540	0.019
	≥11	160	28.1%	136	26.5%		
HADs Anxiety Scale	≤10	291	51.1%	245	47.7%	0.253	0.035
	≥11	278	48.9%	269	52.3%		

* *p* < 0.05.

### 3.3 Multivariate logistic regression for determinants of energy drink

Multivariate logistic regression was performed to investigate the determinants of energy drinks consumption, as shown in Table 4. The findings showed that participants whose father’s education was more than 12 years (AOR: 1.652, *p* < 0.001) were more likely to consume energy drinks than those with an education level equal to or less than 12 years. Furthermore, participants who smoked cigarettes were twice more likely to consume energy drinks than the participant who answered no (AOR: 2.240, *p* < 0.004). Additionally, participants who drank alcohol were twice as likely to consume energy drinks compared to those who answered no (AOR: 2.374, *p* < 0.005). Finally, participants who used waterpipe tobacco smoking were five times more likely to consume energy drinks than those who answered no (AOR: 5.401, *p* < 0.001).

**TABLE 4:** Multivariate logistic regression for determinants of energy drinks.

Characteristics		Energy drinks		Adjusted analysis 95% CI, AOR*	
		Sig.	AOR	Lower	Upper
Father’s education level	>12 years	<0.001	1.652	1.252	2.180
	≤12 years	Reference			
Did you smoke cigarettes in last year?	Yes	0.004	2.240	1.296	3.873
	No	Reference			
Did you drink alcohol in last year?	Yes	0.005	2.374	1.304	4.322
	No	Reference			
Did you use waterpipe tobacco smoking?	Yes	<0.001	5.401	3.658	7.975
	No	Reference			

The multivariate logistic regression model includes study variables: Gender, type of school, academic stream, place of residency, father’s work, mother’s work, father’s education level, mother’s education level, smoke cigarettes, drink alcohol, energy drinks, use of waterpipe tobacco smoking, and take any drugs or substance, HADs Depression Scale and HADs Anxiety Scale. AOR, adjusted odds ratio; 95% CI, 95% confidence interval; Sig., significance level (*p < 0.05).

## 4 Discussion

This is the first study to employ a large-scale school survey to assess the consumption of energy drinks and its correlation with other substance use among 12th-grade students in Palestine. Our research, which utilized a sample of more than 1083 high school students, demonstrated a high prevalence of energy drinks among 12th grade students (52.5%), which is considered higher than that of other studies in the literature review. For instance, a study conducted in the United States of 9,911 adolescents revealed that the prevalence of energy drink consumption increased substantially for adolescents (0.2%–1.4%), young adults (0.5%–5.5%), and middle-aged adults (0.0%–1.2%) from 2003 to 2016 (26). Teijeiro et al. (27) conducted an additional study in Spain that included students aged 14–18 years and found that the prevalence of energy drink consumption in the past 30 days varied from 40.4% (2014) to 47.7% (2023) (27). According to a study conducted in Canada, 34.1% of students consumed energy drinks (2). In the United States, another study showed that 20% of the students consumed energy drinks (28). However, in a 2013 study conducted in 16 European countries, Zucconi et al. estimated that 68% of European adolescents consumed energy drinks in the previous year. This consumption was greater than that of the other two population categories examined, which were adults and children (4). A study conducted in the United Arab Emirates revealed that 20% of students aged 14–18 consumed energy drinks (29). One study done in Egypt found that 38.5% of adolescents consumed energy drinks (30). In Kuwait, 28.3% of university students consumed energy drinks (31). The consumption of energy drinks by school students aged 12–19 years was found to be 45% in Saudi Arabia (32). Energy drinks are consumed by students for various purposes, including as a study aid, to enhance athletic performance, to lose weight, and to provide a rapid energy boost (2, 33). According to a study conducted by Qtait and Alarab (23), students consume energy drinks for four primary reasons: to maintain alertness, to study, to reduce fatigue, and for their pleasant taste (23).

The high prevalence of energy drinks consumption in the current study may be attributed to the fact that energy drinks are acceptable in the Palestinian culture and among the students’ families. Consequently, these students may consume these energy drinks to enhance their energy levels and assist them in completing their schoolwork. Nevertheless, this consumption may expose students to a variety of health problems due to their lack of awareness regarding their potential risks. For example, consumption of energy drinks can have adverse health consequences, including dehydration, arrhythmia, heart failure, anxiety, and insomnia (34–37). Thus, it is imperative to heighten the awareness of 12th grade students, teachers, and their families regarding the hazards of consuming energy drinks. Also, additional research is required to examine the reasons for the high consumption of energy drinks among this age group.

Furthermore, our results indicated that participants whose father’s educational level was more than 12 years were more likely to consume energy drinks, which is consistent with the findings of other studies (38–41). However, our result is in contrary to the findings of other studies. For example, a study conducted in Poland by Mularczyk-Tomczewska et al. (42) revealed that individuals with higher education and a higher financial status were more likely to perceive energy drinks as detrimental and support the ban (42). Additionally, Teijeiro et al. (27) found that students are more likely to consume energy drinks if they have no parent with a higher education (27). One potential explanation for the findings of our study is that fathers with a high level of education will have access to high-paying jobs, which will increase the likelihood that these students will have enough money to purchase these energy drinks (39). This is particularly relevant in Palestine, where parents are responsible for their children’s life expenses until they graduate from university and secure employment. Furthermore, it is possible that parents with higher educational backgrounds may mistakenly view energy drinks as a “legal and accessible tool” for their children to maintain high academic performance under pressure. As a result, these students may be motivated to consume energy drinks to achieve high grades due to increased parental pressure to improve their academic performance. In Korea, Shim and Lee, (43) indicated that highly educated parents with high employment positions are frequently focused on obtaining admission for their children to prestigious colleges. They are exceedingly passionate about their children’s education. Consequently, teenagers from these homes may face an increased pressure for academic achievement, resulting in their regular intake of energy drinks (43). Research indicates that energy drinks may temporarily enhance cognitive functioning; however, these benefits are inconsistent and associated with considerable concerns about negative health consequences (44, 45). Wierzejska et al. (46) found that the majority of students from primary and secondary schools (54%) did not believe that energy drinks could be harmful to their health (46). Therefore, it is essential to help parents and teenagers understand the potentially harmful effects of energy drinks (43).

Furthermore, while our findings did not reveal an association between energy drink consumption and substance use, they did show that participants who smoked cigarettes, drank alcohol, and used cigarettes and waterpipe tobacco were more likely to consume energy drinks, which contradicted the findings of other studies (2, 15, 16, 18, 19, 47). For example, a study conducted in Canada revealed that the consumption of energy drinks among students in middle and high schools was correlated with alcohol use, opioid use, excessive drinking, cannabis use, and tobacco cigarette smoking. Furthermore, the study revealed that 42.1% of adolescents who reported alcohol consumption in the previous year also reported energy drink consumption (2). In Turkey, a study conducted by Evren and Evren showed that the consumption of energy drinks was correlated with a higher risk of chronic tobacco, alcohol, and illicit drug use. The association between substance use and frequent consumption was stronger for energy drink consumption (48). Furthermore, Nordt et al. (33) found that 24% of adolescents who visited the emergency room for any reason reported using energy drinks containing ethanol or narcotics, including cannabis, cocaine, and methamphetamine. Also, they found that 42.1% of adolescents who reported consuming alcohol in the previous 12 months also reported consuming energy drinks in the same period, as opposed to only 18.6% of those who did not report consuming alcohol (33). Other studies, similar to ours, did not observe a correlation between substance use and energy drinks (49).

One potential explanation for the lack of association between energy drinks and substance use is the substitution effect. Shapira et al. (50) define substitution as the deliberate decision to use one drug, whether licit or illicit, in place of another, based on perceptions of cost, availability, safety, legality, substance characteristics, and substance attributions (50). Several factors may motivate substitutions, such as the drug user’s expectation of improved drug purity, increased availability or reduced cost of the substitute drug, or positive expectancies in general regarding the substitute drug’s effects (51). For instance, someone may employ substances such as marijuana, crack, cocaine, hashish, and heroin as their primary medications to alleviate social anxiety or relax, thereby eliminating the necessity for energy beverages’ stimulating effects. Furthermore, the substitution of energy drinks for more hazardous or illegal substances may be due to the fact that these substances are less acceptable in these households than energy drinks and are classified as illegal. Additionally, underreporting may be a contributing factor, as this is a sensitive subject that is challenging to address in an anonymous survey. For example, Steinhoff et al. (52) revealed that the prevalence of the majority of substances was underreported by 30%–60% of young adult. They also indicated that young persons may be hesitant to disclose their illicit substance use due to concerns regarding the legal or societal consequences (52).

Additionally, in instances where the availability of products or local norms differs from those of other countries, cultural or geographical norms may offer further explanation. For example, the participants in our study may not decide to purchase an energy drink in order to reduce the cost, as the Palestinian government imposes an excessive tax on alcohol and tobacco. Consequently, it is essential that school environments establish well-organized psychological interventions and awareness initiatives to inform 12th-grade students about the risks associated with substance use. It is important that these programs address all students, regardless of whether they consume substances, to promote their health and reduce their consumption of energy drinks. Finally, the current study found no association between energy drinks and depression or anxiety, which is consistent with other studies (53, 54) and contradicts other previous findings (3, 20, 21). According to Snipes et al. (53), people who have high anxiety may avoid energy drinks since their stimulant qualities have the potential to aggravate their symptoms (53). One potential explanation is that individuals who experience low moods or emotional disorders, such as anxiety and depression, may self-medicate by consuming energy drinks as a temporary “pick-me-up.” Consequently, this utilization pattern may explain the reason why the transient mood effects are frequently positive, while the long-term associations are not. This concept is supported by the fact that caffeine is frequently employed by students as a coping mechanism during stressful circumstances (21).

### 4.1 Strengths and limitations

Our study has several limitations. The data depended on self-reports, which may be susceptible to recall bias. Also, the data concerning energy drink use and drug use were self-reported, particularly were sensitive questions, which may have led participants to underreport or exaggerate their responses. Future studies are necessary to examine in-depth the differences in the consumption of alcohol, smoking, and substance use with energy drinks. Further, the study employed a cross-sectional design; hence, it cannot determine causal associations between energy drink consumption and substance use. Therefore, longitudinal studies are necessary to assess the association between them. In addition, the majority of the participants in the current study are females. Consequently, additional research is necessary to recruit more males in order to examine the differences between the two groups in relation to energy drinks. Furthermore, the use of convenience sampling may limit the generalizability of the findings and reduce their representativeness. Also, in the current study, we asked about energy drink intake during the last month; thus, future research may investigate consumption throughout the past year and assess reasons behind students’ use of these energy drinks. Moreover, our results did not reveal a significant link between depression, anxiety, and energy drink intake; consequently, future research can examine other factors, such as stress, and academic pressure. Notwithstanding these constraints, the findings of the current study provide important data about the psychological well-being of adolescents living in war-affected areas. This study significantly contributes to the literature by being the first to assess the relationship between consumption of energy drinks and other mental problems such as depression, anxiety, substance use, alcohol intake and smoking among 12th-grade adolescents in a developing country experiencing armed conflict and political violence.

### 4.2 Implication for practice

The findings of our study indicate that the consumption of energy drinks is elevated among 12th grade students, who are under significant academic pressure to succeed in the Tawjihi Exam (General Secondary Education Certificate Test). Our findings emphasize the necessity of increasing adolescent awareness and educating them on the detrimental effects of energy drink consumption. This illustrates the value of educating students about the hazards of energy drinks, modifying their inaccurate and perilous beliefs regarding their utility during academic or leisure activities, and increasing their awareness of the risks associated with their consuming behavior. Students need education on how to engage in alternative coping strategies for managing academic performance. Regulation of advertising and the implementation of purchase restrictions are essential to control their consumption of energy drinks. We may also inform teachers, parents, and health service providers about the negative and adverse effects of these students’ energy drink consumption. This study’s significant finding is the absence of a positive correlation between substance use and energy drinks. Thus, these students may perceive these behaviors as substitutes rather than complements. Prevention programs may require modification to address each behavior separately rather than a combined approach. Additionally, households with a high level of education and income may require education and awareness regarding the potential hazards associated with their children’s consumption of energy drinks. Also, students who smoke and consume alcohol are more likely to consume energy drinks. So, energy drink consumption trends and user characteristics have to be analyzed by the Palestinian Ministry of Education and Higher Education to establish effective regulations in schools and develop targeted information campaigns. Finally, in order to confirm these results and evaluate the correlation between energy drink consumption and substance use, smoking, alcohol, depression, and anxiety among 12th grade students, additional longitudinal research is required.

## 5 Conclusion

The findings of our study indicate that the consumption of energy drinks is high among 12th-grade students. Students who smoke and consume alcohol are more likely to consume energy drinks. Our findings emphasize the need to increase adolescents’, families’, and teachers’ awareness and educate them about the detrimental effects of energy drink consumption.

## Data Availability

The original contributions presented in this study are included in this article/supplementary material, further inquiries can be directed to the corresponding author.
